# Why monitor the neonatal brain—that is the important question

**DOI:** 10.1038/s41390-022-02040-9

**Published:** 2022-04-01

**Authors:** Sampsa Vanhatalo, Nathan J. Stevenson, Ronit M. Pressler, Nicholas S. Abend, Stéphane Auvin, Francesco Brigo, M. Roberta Cilio, Cecil D. Hahn, Hans Hartmann, Lena Hellström-Westas, Terrie E. Inder, Solomon L. Moshé, Magda L. Nunes, Renée A. Shellhaas, Kollencheri P. Vinayan, Linda S. de Vries, Jo M. Wilmshurst, Elissa Yozawitz, Geraldine B. Boylan

**Affiliations:** 1 BABA Center in Pediatric Research Center, Departments of Clinical Neurophysiology and Physiology, Children’s Hospital and HUS Imaging, Helsinki University and Helsinki University Hospital, Helsinki, Finland; 2 Brain Modelling Group, QIMR Berghofer Medical Research Institute, Brisbane, QLD Australia; 3 Clinical Neuroscience, UCL GOS Institute of Child Health, Department of Clinical Neurophysiology, Great Ormond Street Hospital for Sick Children, London, UK; 4 Division of Neurology and Neurophysiology Lab, Children’s Hospital of Philadelphia, Philadelphia, PA USA; 5 Université de Paris, Service de Neurologie Pédiatrique, Hôpital Universitaire Robert Debré, APHP, Institut Universitaire de France (IUF), Paris, France; 6 Department of Neurology, Hospital of Merano (SABES-ASDAA), Merano, Italy; 7 Division of Pediatric Neurology, Cliniques universitaires Saint-Luc, Université catholique deLouvain, Brussels, Belgium; 8 Division of Neurology, The Hospital for Sick Children and Department of Paediatrics, University of Toronto, Toronto, ON Canada; 9 Clinic for Pediatric Kidney, Liver and Metabolic Disorders, Hannover Medical School, Hannover, Germany; 10 Department of Women’s and Children’s Health, Uppsala University, Uppsala, Sweden; 11 Department of Pediatric Newborn Medicine, Brigham and Womens Hospital, Harvard Medical School, Boston, MA USA; 12 Isabelle Rapin Division of Child Neurology, Saul R. Korey Department of Neurology, and Departments of Neuroscience and Pediatrics, Albert Einstein College of Medicine and Montefiore Medical Center, Bronx, NY USA; 13 Brain Institute (BraIns), Pontifical Catholic University of Rio Grande do Sul, Porto Alegre, Brazil; 14 Department of Pediatrics, Division of Pediatric Neurology, University of Michigan, Ann Arbor, MI USA; 15 Department of Pediatric Neurology, Amrita Institute of Medical Sciences, Cochin, Kerala India; 16 Department of Neonatology, University Medical Center Utrecht, Utrecht University, Utrecht, the Netherlands; 17 Department of Paediatric Neurology, Red Cross War Memorial Children’s Hospital, Neuroscience Institute, University of Cape Town, Cape Town, South Africa; 18 Isabelle Rapin Division of Child Neurology, Saul R. Korey Department of Neurology, Montefiore Medical Center, Albert Einstein College of Medicine, Bronx, NY USA; 19 INFANT Research Centre & Department of Paediatrics & Child Health, University College Cork, Cork, Ireland

## Abstract

A key goal of neonatal neurocritical care is improved outcomes, and brain monitoring plays an essential role. The recent NEST trial^1^ reported no outcome benefits using aEEG monitoring compared to clinical seizure identification among neonates treated for seizures. However, the study failed to prove the effects of monitoring on seizure treatment in the first place.

Neonates with acute neurological disorders, such as encephalopathy due to hypoxia-ischemia, will commonly undergo continuous electroencephalography (EEG) monitoring to assess brain recovery and detection of seizures. EEG monitoring is necessary for a reliable diagnosis and monitoring of neonatal seizures^2^, because the majority of EEG-confirmed neonatal seizures have no clinical signs, while EEG monitoring may confirm that many clinical events are non-epileptic in origin^3^. The International Neonatal Consortium, European Medicines Agency, US Food and Drug Administration, Brighton Collaboration, the International League Against Epilepsy, and American Clinical Neurophysiology Society agree that all studies involving the treatment of neonatal seizures should evaluate seizures from the EEG recordings^2,4,5^.

Before these international recommendations, it was common for clinicians to question the added value of (a)EEG monitoring in newborn care. The NEST trial^1^ attempted to address this question by assessing whether neurodevelopmental outcomes are improved by treating seizures with the aid of amplitude-integrated EEG monitoring (aEEG) compared to treating seizures identified by clinical recognition only. The underlying assumption was that seizure identification and management reduces secondary brain injury and improves neurodevelopment (Fig. 1), and that aEEG facilitates seizure diagnosis and treatment. The study found no evidence of improved neurodevelopmental outcomes, and secondary analyses showed reduced cognitive outcomes in the group with aEEG monitoring. The secondary outcome findings can be linked to many well-established confounders, only some of which were mentioned in the paper. However, the most surprising finding of this study is the lack of difference in overall seizure burden between groups treated with aEEG monitoring support versus those with clinical consideration only.

**Fig. 1:**
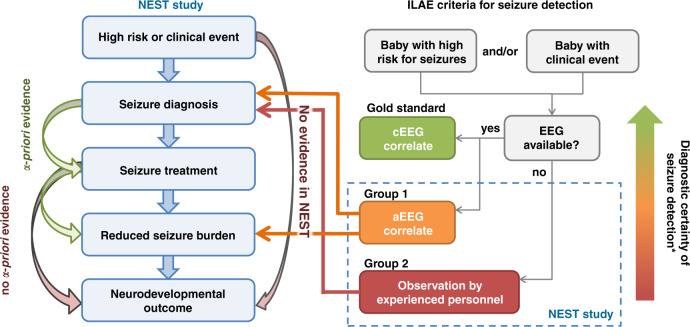
The left side flowchart summarizes rationale in the NEST study and its relationship with the international guideline of seizure detection (right side flowchart). Colors from red through orange to green code for established reliability in seizure detection. The arrows depict the assumed causal links from the clinical suspicion to the neurodevelopmental outcome. The green arrows on the left side show links with prior research evidence. The gray arrow shows the critical a priori missing evidence that should have been known before commencing the NEST study. Arrows on the right side show the links that were examined in the NEST with negative findings. Notably, NEST study relies on seizure detection by aEEG that only has modest- or low-level reliability. This effectively precludes the potential to use NEST design for examining its study question. *Diagnostic certainty according to Brighton Collaboration case definition of neonatal seizures. Level 1—gold standard, definite seizure (seizures confirmed on EEG with or without clinical manifestations); level 2—probable seizure (aEEG confirmed or clinically assessed focal clonic or focal tonic seizures); level 3—possible seizure (clinical events suggestive of epileptic seizures other than focal clonic or focal tonic seizures); level 4—not seizure (reported clinical events that do not meet case definition); level 5—not seizure (clinical events evaluated by EEG and diagnosed as not a seizure).

It is now widely recognized that (a)EEG monitoring and seizure treatment should commence as early as possible because the seizure burden peaks during the first 24 h in infants with hypoxic-ischemic encephalopathy (the primary cause of neonatal seizures) with subsequent gradual resolution^6,7^. This spontaneous resolution of seizures over time means that a late start of treatment would require prohibitively large study cohorts and only show a treatment response with limited clinical significance^8^. In the NEST study, aEEG was initiated after 24 h; therefore, NEST likely missed the bulk of the seizure burden, and it only assessed the remains of the spontaneously decaying seizures. Thus, the NEST study design per se precluded an opportunity to see an actual treatment effect in either study arm^8^.

In addition to missing seizures due to the late onset of aEEG monitoring, the EEG assessment method per se used in the NEST study introduces a major confounder in seizure burden estimates (Fig. 1). Although aEEG is often useful for clinical brain monitoring, including the recognition of longer seizures, international guidelines have established that the aEEG alone cannot provide accurate enough estimates of seizure burden. The NEST study amplifies this bias by only considering the longer seizures that were evident on the compressed aEEG trace that likely led to a considerable proportion of the seizure burden going undetected, untreated, and not accounted for in the analyses. Moreover, the report does not provide key details regarding the aEEG, for instance, which recording channels were used, how aEEG review was actually done at the bedside, whether seizure detection algorithms were (or were not) used, when treatment(s) were administered, and how seizure burden and response to treatment were subsequently quantified. Furthermore, analyses of aEEG background patterns are not reported despite their strong associations with outcome^9^.

Overall, the NEST study portrays a grand challenge in all contemporary medical research. The rapidly changing clinical landscape and accumulating research data make it difficult to design new studies with influential results. This is particularly true for neonatal research where the use of long-term neurodevelopment outcomes results in very long study cycles. Consequently, the initial study question may have lost its relevance by the time the trial is completed. In the case of NEST, the concurrently accumulated evidence rendered the initial study question a classic straw man hypothesis: The study was designed to test the hypothesis that aEEG improves neurodevelopmental outcomes by improving seizure identification and seizure management, thereby reducing seizure burden (Fig. 1). Instead, the study shows that aEEG begun >24 h after delivery did not affect treatment practices and outcomes, but it does not really address the question of whether or how early use of EEG monitoring impacts treatment success, i.e., seizure burden en route to better outcomes. Notably, this situation is changing rapidly worldwide, with further training of personnel as well as more widespread use of EEG devices with adequately validated algorithms for automated seizure detection^10^.

Why is this issue important to raise? We believe that there is a risk that the NEST study question per se may lead clinicians to erroneously conclude that EEG monitoring is unhelpful in neonatal neurological management. This misleading effect may be worse in low- and middle-income countries where the current upskilling of neonatal units is calling for de novo implementation of brain monitoring routines. Notably, the authors of NEST study concluded that “EEG remains the reference standard for the detection of neonatal seizures, and essential in the validation of neonatal research.” This important statement carries the readership onto the grand challenge question of neonatal neurological care: Why monitor the neonatal brain? Routine clinical practice has shown that EEG monitoring of neonates supports and guides individualized patient care by providing a functional measure of brain recovery after injury, reliable detection of seizures, and surveillance of sleep-wake cycling, as well as many other context-relevant observations^9–11^. In addition, several novel aEEG and EEG biomarkers are being developed that hold promise for making EEG a proximal outcome measure for individualized patient care, benchmarking clinical trials, as well as predicting later neurodevelopmental compromise. These benefits are irrefutable and widely acknowledged, and conceivably, trying to measure their links to neurodevelopmental outcomes without considering the possible intermediary or causal mechanisms is unproductive. Indeed, there is a widely shared bedside experience in neonatal intensive care units that trusts in a well-known mechanism, the latent Hawthorne effect^11,12^: mere attention to the newborn brain leads to improved newborn care.

## References

[1] Hunt RW (2021). Effect of treatment of clinical seizures vs electrographic seizures in full-term and near-term neonates: a randomized clinical trial. JAMA Netw. Open.

[2] Soul JS (2019). Recommendations for the design of therapeutic trials for neonatal seizures. Pediatr. Res..

[3] Malone A (2009). Interobserver agreement in neonatal seizure identification. Epilepsia.

[4] Pressler RM (2021). The ILAE classification of seizures and the epilepsies: modification for seizures in the neonate. Position paper by the ILAE Task Force on Neonatal Seizures. Epilepsia.

[5] Pellegrin S (2019). Neonatal seizures: case definition & guidelines for data collection, analysis, and presentation of immunization safety data. Vaccine.

[6] Pavel AM (2021). Neonatal seizure management: is the timing of treatment critical?. J. Pediatr..

[7] Rennie JM (2019). Characterisation of neonatal seizures and their treatment using continuous EEG monitoring: a multicentre experience. Arch. Dis. Child Fetal Neonatal Ed..

[8] Stevenson NJ, Boylan GB, Hellstrom-Westas L, Vanhatalo S (2016). Treatment trials for neonatal seizures: the effect of design on sample size. PLoS One.

[9] van Rooij LG (2005). Recovery of amplitude integrated electroencephalographic background patterns within 24h of perinatal asphyxia. Arch. Dis. Child Fetal Neonatal Ed..

[10] Pavel AM (2020). A machine-learning algorithm for neonatal seizure recognition: a multicentre, randomised, controlled trial. Lancet Child Adolesc. Health.

[11] Glass HC (2010). Neurocritical care for neonates. Neurocritical Care.

[12] McCarney R (2007). The Hawthorne effect: a randomised, controlled trial. BMC Med. Res. Methodol..

